# Life satisfaction among a clinical eating disorder population

**DOI:** 10.1186/s40337-020-00326-z

**Published:** 2020-10-13

**Authors:** Elizabeth A. Claydon, Caterina DeFazio, Christa L. Lilly, Keith J. Zullig

**Affiliations:** 1 Department of Social and Behavioral Sciences, West Virginia University School of Public Health, One Medical Center Drive, P.O. Box 9190, Morgantown, WV 26506-9190 USA; 2 Department of Biostatistics, West Virginia University School of Public Health, Morgantown, WV USA

**Keywords:** Life satisfaction, Eating disorders, Quality of life, Clinical populations

## Abstract

**Background:**

The primary objective was to understand life satisfaction (LS) of patients with eating disorders (EDs) in relation to eating pathology severity, personal/familial ED history, and key demographic and anthropometric variables.

**Methods:**

Participants (*N* = 60) completed the Satisfaction with Life Scale (SWLS), the Eating Pathology Severity Index (EPSI), and demographic questionnaires. Bivariate associations via correlations and multiple linear regression models were used to explore these relationships.

**Results:**

The SWLS mean score was 3.7 out of 7, suggesting it is below the population-based norm. LS was positively statistically significantly associated with private insurance, past ED, EPSI muscle building, EPSI restricted eating, and EPSI negative attitudes. When included in multiple linear regression, the model explained 33% of the variability of LS [F (7, 56) = 3.4, *p* = 0.0054, R^2^ = 0.33]. EPSI muscle building remained the strongest predictor (β = 0.13, *p* = 0.04).

**Conclusions:**

Based on the data, individuals who have/have had EDs scored lower on the SWLS than the general population. Individuals scoring within this range typically experience significant issues in several areas of life or a substantial issue in one area.

## Plain language summary

The goal of this study was to assess life satisfaction of individuals with eating disorder diagnoses. We also looked at the connection of their life satisfaction with the severity of their symptoms, family history of eating disorders, and other personal characteristics. For this study, we collected questionnaires from 60 participants who had a clinical diagnosis of an eating disorder. Our analyses showed that sample mean life satisfaction was below population-based norms and was significantly associated with private insurance, a past eating disorder, and some specific eating disorder symptoms. These findings can help inform interventions that focus on both improving life satisfaction and eating disorder symptoms.

## Background

Promoting healthy development, healthy behaviors, and well-being across all life stages is a primary overarching Healthy People 2030 goal from the United States (US) Department of Health and Human Services [14]. These goals suggest that methods for improving quality of life (QOL) remain a priority for reaching 2030 goals. QOL has been operationalized, measured, and applied in interventions from both objective and subjective perspectives. Objective QOL indicators focus on population-based criteria associated with “good” lives such as income level, access to health care, etc. [3]. However, life satisfaction (LS) is frequently used as an subjective indicator of QOL owing to its ability to extend beyond transient affective life experiences and include an evaluative and reflective appraisal of life overall [6, 11, 23, 24]. In addition, because research with adults demonstrates that objective and subjective indicators (e.g., physician versus self-ratings of health) are distinct from each other, researchers have suggested that objective and subjective QOL indicators reflect separate, but complementary information [10]. This study focuses on LS, which Diener [6] defines as the cognitive assessment an individual makes regarding their feelings and attitudes relative to their life at the time.

LS research is receiving increased interest owing to associations with numerous health conditions such as asthma, diabetes, obesity, arthritis, and heart disease [26]. For example, one study which utilized the Satisfaction with Life Scale (SWLS) found that negative LS was related to anxiety and depression where positive LS has been shown to be significantly related to successful life adaptation [18]. However, LS among individuals with EDs remains relatively underexplored, which may be a barrier in providing effective care and preventing relapse [16]. This is surprising given research suggesting improved QOL is related to better ED treatment outcomes, including motivation to change one-year post treatment [20], and reductions in ED psychopathology, depression, and anxiety 6-months post treatment [17].

EDs carry high mortality risks [25] and are more frequently diagnosed in young women than men [22]. The three most common EDs are anorexia nervosa (AN), bulimia nervosa (BN), binge eating disorder (BED), and Other Specified Feeding or Eating Disorders (OSFED) [2, 15]. According the DSM-5 (APA, 2013), AN is characterized by an irrational fear of gaining weight, distorted body image, excessive food restriction, amenorrhea, and weight issues (< 85% of clinically standard weight). BN is characterized by bingeing and purging and typically measured by those who may engage in excessive exercise, vomiting, laxatives, and/or diuretics use. BED has similar binges (large amount of food within any 2 h period of time), but is not followed by compensatory behavior (APA, 2013). Those with BED also express a loss of control and distress during the binge episodes. Finally, OSFED is a category reserved for those EDs which do not meet the criterion for AN or BN.

To our knowledge, there is little literature exploring the association between LS and EDs in a clinical population. In a Spanish study by Magallares et al. [18], the relationship between anorexia nervosa (AN), bulimia nervosa (BN), Other Specified Feeding and Eating Disorders (OSFED), and Subjective Well-Being (SWB; includes positive and negative affect as well as LS) was explored. This study found women with EDs reported lower SWB than those in the control group. Kitsantas et al. [16] found college students diagnosed with EDs reported significantly lower levels of LS as well as higher levels of negative affect than those at-risk (do not meet ED criteria without more in-depth clinical evaluation) for EDs or at normal weight. Other research indicates individuals who have fully recovered from AN have similar LS scores to healthy controls, while those who have poor outcomes at the end of treatment have significantly lower LS [13] and QOL.

The current study seeks to improve the extant literature by utilizing the well-validated and widely used Satisfaction with Life Scale (SWLS [7]) and draws from a clinical population in four different US states allowing geographic diversity. The primary study aim was to understand LS in a clinical population of patients with ED diagnoses. Based on the results by Magallares et al. [18], we hypothesized that individuals in this population would report lower LS than population-based norms without an ED diagnosis also using the SWLS. Exploratory study aims were to examine subgroup analyses to understand the association between LS and other demographic characteristics such as ED history, insurance coverage, BMI, and income level for the first time. Such analyses are expected to provide treatment providers and researchers descriptive data to help improve treatment outcomes for those with EDs.

## Methods

### Data collection

Participants completed the online surveys that were advertised through four distinct ED treatment sites: The Eating Recovery Center in Cincinnati, Ohio; The University of North Carolina Center of Excellence for Eating Disorders; The Chestnut Ridge Center in Morgantown, West Virginia; and the eating disorders partial hospitalization program (PHP) at New York-Presbyterian Hospital (methods described in previous publication [4]). The survey was also advertised with permission through the West Virginia Eating Disorder Network.

### Participants

A total of 65 participants completed the online survey between January and March of 2017 through advertisements posted and shared in clinics. This sample was chosen for a previous study and was powered based on findings from previous research in a community sample indicating a sample size of 58 participants was adequate [4, 5]. Participants were included in the analysis if they provided demographic information, completed the SWLS, and completed the EPSI, reducing the total sample to 60. Additional detail regarding participant recruitment has been published previously [4].

### Measures

The *Eating Pathology Symptom Inventory (EPSI)* was provided to assess ED symptoms [12]: This inventory contains a total of 45 Likert scale items (ranging from *Never* to *Very Often*) with eight separate subscales: Body Dissatisfaction, Binge Eating, Cognitive Restraint, Purging, Restricting, Excessive Exercise, Negative Attitudes towards Obesity, and Muscle Building. The EPSI displays excellent estimates of validity, internal consistency (α = 0.84–0.89) and test-retest reliability (Pearson *r* = 0.73 [12]). Within our sample, we found high internal reliability (α = 0.93).

*Satisfaction with Life Scale* (SWLS [7, 8]): This scale consists of five items measuring a participant’s satisfaction with life using a Likert (1–7) scale. A global score is calculated by summing the scores from the five items. The internal reliability of the SWLS in our sample was high, (α = 0.92).

*Self-Report ED Diagnoses –* As part of the survey, participants provided self-report data about both past and present ED diagnoses in response to the question: “*What eating disorders have you had (select all that apply)?”* They could then select from a list and include more than one diagnoses for both current and past, including from a list of: anorexia, bulimia, binge eating disorder, and other specified feeding and eating disorder. There were also options for each disorder to specify whether the diagnosis was a professional diagnosis or self-diagnosis.

### Human subjects

This study was filed with the referent university’s Institutional Review Board and exempt status was acknowledged (IRB#: 1609282716). This exempt status was granted because signed informed consent was waived due anonymous survey completion. However, a cover letter explaining the study was presented prior to the survey and all participants had to select that they agreed to participate in order to continue to the survey. Qualtrics software was used to both host and distribute the survey online. Additionally, following the survey, a list of resources and referrals for ED treatment and support groups for all individuals. No protected health information (PHI) was obtained. A copy of the complete survey was published previously [4].

### Data analysis

Data were described using frequencies and valid percentages for categorical variables, and means and standard deviations for continuous variables. To answer the primary research question, bivariate Pearson correlations were run to understand the relationship between variables. Then multivariable ordinary least square linear regressions using stepwise selection were run with predictors of mean LS scores. An initial model for mean LS was run with the covariates of any past ED and private insurance only, and then a second model was run with those covariates as well as the EPSI subscales which had bivariate associations with *p*-values smaller than 0.1 (these subscales were: EPSI Body Dissatisfaction, Restricted Eating, Negative Attitudes toward Obesity, Muscle Building, and Purging. This conservative alpha (0.10) was chosen for multivariable model inclusion consistent with a purposeful selection process in order to reduce the number of variables included in the model while preserving likely significant relationships. All data analyses were conducted using SAS JMP® 14.0.

## Results

### Descriptive statistics

Most participants were white (96.7%) and female (93.3%). The mean age was 31.8 (SD = 9.9) years and the majority reported a college education or higher (78.3%) and a past ED diagnosis (73.3%). Other sample demographic characteristics are located in Table 1. The mean SWLS score for the sample was 3.7 (SD = 1.6) out of 7, suggesting below population-based LS norms [8].

**Table 1:** Demographic Characteristics (*N* = 60)

Characteristic	N (%)	M (SD)
**Life Satisfaction (avg.)**		3.7 (1.6)
**Age**		31.8 (9.9)
**Gender**		
Male	4 (6.7)	
Female	56 (93.3)	
**Race/ethnicity**		
White	58 (96.7)	
Other	2 (3.3)	
**College Education or Higher**		
Yes	47 (78.3)	
No	13 (21.7)	
**Annual Family Income**		
< $46,000	14 (23.3)	
$46,000 - $100,000	21 (35.0)	
> $100,000	18 (30.0)	
Prefer not to answer	7 (11.1)	
**Parent**		
Yes	13 (21.3)	
No	47 (78.7)	
**Private Insurance**		
Yes	45 (75.0)	
No, Medicare	15 (25.0)	
**Any Past Eating Disorder**		
Yes	44 (73.3)	
No	16 (26.7)	
**Any Current Eating Disorder**		
Yes	34 (56.7)	
No	26 (43.3)	
**BMI**^**a**^		22 (6.8)
Underweight	13 (22.4)	
Healthy Weight	36 (62.1)	
Overweight	3 (5.2)	
Obese	6 (10.3)	
**EPSI - Body Dissatisfaction**		25.3 (6.5)
**EPSI - Restricted Eating**		15.7 (6.0)
**EPSI - Cognitive Restraint**		10.1 (3.4)
**EPSI - Purging**		10.4 (6.6)
**EPSI - Excess Exercise**		13.0 (6.5)
**EPSI - Negative Attitudes**		11.9 (6.6)
**EPSI - Muscle Building**		7.6 (3.5)
**EPSI - Binge Eating**		17.9 (8.6)

^a^ Using CDC BMI categories

### Bivariate and inferential statistics

LS was statistically significantly associated with two demographics: private insurance (*r* = 0.36, *p* = 0.008) and past ED (*r* = 0.36, *p* = 0.008). No other demographic variables were significantly associated with LS. LS was significantly correlated with the following EPSI subscales: EPSI muscle building (*r* = 0.28, *p* = 0.044), EPSI restricted eating (*r* = − 0.28, *p* = 0.044), and EPSI negative attitudes (*r* = − 0.27, *p* = 0.054). EPSI body dissatisfaction (*r* = − 0.25, *p* = 0.067) and EPSI purging (*r* = − 0.23, *p* = 0.091) were also included in the model as the bivariate associations were below the a-priori determined alpha of 0.10.

When all variables with bivariate statistical significance below the a-priori alpha were included in a multiple linear regression, the model (Model 2 in Table 2) explained 33% of the variability in LS [F (7, 56) = 3.4, *p* = 0.0054, R^2^ = 0.33]. EPSI muscle building remained the strongest and only statistically significant predictor (β = 0.13, *p* = 0.04). While demographic and sample characteristics of past ED and private insurance explained 24% of the variability in LS, the EPSI subscale explained an additional 9% variability in LS.

**Table 2:** Multivariable linear regression using stepwise regression with predictors of average life satisfaction. The second model includes covariates and bivariate associations. (*N* = 56)

	Model 1				Model 2			
	Covariates				Covariates + Bivariate Associations > .1			
**Overall Model**	**F**	**df**	**p**	**R**^**2**^	**F**	**df**	**p**	**R**^**2**^
	8.9	2	.0004*	0.24	3.4	7	.0054*	0.33
**Predictors of Avg. Life Satisfaction Score**	**ß** ***±*** **SE**		**p**			**ß** ***±*** **SE**		**p**
Intercept	1.93 *±* 0.45		<.0001**			2.81 *±* 1.16		0.0191
Any Past ED	1.17 *±* 0.41		0.006*			0.83 *±* 0.45		0.072
Private Insurance	0.19 *±* 0.36		.0059*			0.92 *±* 0.50		0.074
EPSI Body Dissatisfaction						−0.04 *±* 0.03		0.2738
EPSI Restricting						−0.0005 *±* 0.04		0.9898
EPSI Negative Attitudes						−0.01 *±* 0.04		0.7302
EPSI Muscle Building						0.13 ± 0.06		0.0419*
EPSI Purging						−0.02 ± 0.04		0.5486

* *p* < .05

** *p* < .0001

## Discussion

Preliminary results support our hypothesis that individuals with EDs would have lower LS than those in the general public. Pavot and Diener [21] state that a sum score of 20 (*M* = 4.00) is considered neutral. Magallares et al. [18] found women with EDs had lower SWB (*M* = 3.39–3.97) scores than the control group (*M* = 5.08). According to Maltby and Day [19], English adults reported a mean SWLS sum score of 23.0 (*M* = 4.6) for men and 23.7 (*M* = 4.74) for women. The mean SWLS score in our study sample was 3.7. Therefore, our results are consistent with Magallares et al.’s [18] finding. These results are not necessarily unexpected given that negative attitudes, body dissatisfaction, restricted eating, and purging were expected to have a negative correlation with LS. However, novel findings from the exploratory analysis suggest that past EDs and having private insurance are positively correlated to LS. Although somewhat speculative, a possible explanation for this finding with past ED could be that these individuals have moved beyond initial resistance to treatment because they recognize the severity of their ED and no longer hide their symptoms from others. Thus, this self-awareness may make these individuals less refractory toward seeking professional help when symptoms of the ED recur [28]. From a resources theory viewpoint [9], individuals with a greater number of assets (economic, social, and personal) are better able to meet their needs compared to those with fewer assets. Previous EDs and potential associated increased family social support may provide greater personal self-awareness possibly providing an explanation for our finding between previous EDs and increased LS. Additionally, the positive relationship between private insurance and LS may be partially explained by access to increased treatment options. The insurance structure in the United States is such that private versus public insurance (which was modeled in our study) can be used as an indicator of socioeconomic status (SES).

An unexpected finding was that muscle building was the strongest positive predictor of LS even after accounting for multiple covariates and other ED correlates. Sample questions from the EPSI muscle building subscale include “I used muscle building supplements,” “I thought my muscles were too small,” and “I thought about taking steroids as a way to get more muscular.” One could interpret this through the lens that muscle building is sometimes viewed positively in the ED culture. For example, fads in popular media such as “strong is the new beautiful” [27] idolize the idea that muscle building is good and not pathological. Nevertheless, this preliminary finding would need to be substantiated in other clinical ED samples.

### Limitations

This study is subject to limitations. First, the results from our cross-sectional study design cannot be considered causal. A longitudinal study would assist in clarifying the associations found. Second, the small, clinical sample and convenience sampling limits generalizability, however, this is a traditionally difficult population to access. There were a greater number of females (*N* = 56) than males (*N* = 4) and based on the significance found between the SWLS and the EPSI muscle building scores, future research could benefit from collecting data from an equal proportion of males and females to gain a deeper understanding of this association and how it could possibly relate to sex. Finally, we did not collect information on the duration of illness related to EDs, which would have been helpful to have in relation to LS.

### Strengths

The data was collected from a clinical population which, apart from the study conducted by Magallares et al. [18] Spain, has not been researched in relation to LS and EDs in the United States. Thus, our study helps to fill a previous gap in the literature. Our study had some geographical diversity within the United States also, with participants from West Virginia, Ohio, North Carolina, and New York. This data can assist in providing information that has the potential to be an asset to clinical work. Another strength is that the SWLS and EPSI are widely used, validated scales that can be used in future replication studies with this population.

## Conclusion

Results from this study suggest that clinical ED populations experience lower LS than those in the general population. Additionally, those who reported a previous ED diagnosis and private insurance coverage were likely to have higher LS, suggesting important personal, social and economic resources may impact LS. Results also suggest future research should explore the positive association with LS and EPSI muscle building to gain a more comprehensive understanding of how this measure is being interpreted by participants. Overall, additional research is needed about clinical ED populations’ LS. However, this study has potential implications for care and suggests that attending to one’s LS may be a consideration in ED treatment along with traditional treatments focused on symptom reduction because even small changes could result in increased LS. For example, strategies to increase positive affect (and thus LS) like self-acceptance and personal development/relationships with others, may work synergistically by reducing depression and anxiety [1].

## Data Availability

The datasets used and analyzed as well as code used during the current study are available from the corresponding author on reasonable request.

## Abbreviations

AN: Anorexia
BMI: Body Mass Index
BN: Bulimia
DSM-5: Diagnostic and Statistical Manual of Mental Disorders - 5
ED(s): Eating Disorder(s)
EPSI: Eating Pathology Severity Index
LS: Life Satisfaction
PHI: Protected Health Information
OSFED: Other Specified Feeding or Eating Disorders
QOL: Quality of Life
SES: Socioeconomic Status
SWB: Subjective Well-Being
SWLS: Satisfaction with Life Scale
